# Clinical Manifestations of Behçet’s Disease: A Retrospective Cross-Sectional Study

**DOI:** 10.31138/mjr.34.1.53

**Published:** 2023-03-31

**Authors:** Alireza Sadeghi, Mina Rostami, Ghazaleh Amraei, Fereydoun Davatchi, Farhad Shahram, Arezoo Karimi Moghaddam, Zhaleh Karimi Moghaddam, Alireza Zeraatchi

**Affiliations:** 1 Department of Internal Medicine, Vali-e-Asr Hospital, School of Medicine, Zanjan University of Medical Sciences, Zanjan, Iran,; 2 Social Determinants of Health Research Center, Zanjan University of Medical Sciences, Zanjan, Iran,; 3 Behçet’s Disease Unit, Rheumatology Research Center, Shariati Hospital, Tehran University of Medical Sciences, Tehran, Iran,; 4 Department of Ophthalmology, School of Medicine, Vali-E-Asr Hospital, Zanjan University of Medical sciences, Zanjan, Iran,; 5 Department of Radiation Oncology, Vali-e-Asr Hospital, School of Medicine, Zanjan University of Medical Sciences, Zanjan, Iran,; 6 Department of Emergency Medicine, Valiasr-e-Asr Hospital, Ayatollah Mousavi Hospital, School of Medicine, Zanjan University of Medical Sciences, Zanjan, Iran

**Keywords:** Behçet’s disease, systemic vasculitis, clinical manifestations, anterior uveitis

## Abstract

**Introduction:**

Behçet’s Disease (BD) is a systemic vasculitis, highly prevalent in Eastern Asia to Mediterranean countries. Iran is among the countries with the highest prevalence of BD, and previous studies in different countries have shown a broad range of clinical manifestations of the disease. The present study is conducted to evaluate the prevalence of the clinical manifestations of BD in patients referring to rheumatology clinics of two distinct referral hospitals in Tehran and Zanjan, Iran.

**Methods:**

In this retrospective, cross-sectional study, the medical records of patients with BD were reviewed, and age at onset, sex, the delay between the onset of symptoms and diagnosis, clinical manifestations, HLA B27, HLA B51, HLA B5, haematuria, proteinuria, leukocyturia, Erythrocyte Sedimentation Rate (ESR), and pathergy phenomenon were included in the study. The collected data were analysed by χ^2^ test using SPSS 23.

**Results:**

A total of 188 patients (Male/female ratio = 1.47) were included in the study with mean ± SD age at onset of 27.98 ± 10.47 years and a mean ± SD of delay between the onset of symptom and diagnosis of 5.70 ± 7.16 years. The most common clinical manifestation was mucosal involvement (85.1%), followed by the ocular lesion (55.3%) and skin manifestations (44.7%). The Pathergy phenomenon was observed in 98 patients (52.1%). Moreover, 45.2% had positive HLA B5, followed by HLA B51 (35.1%) and HLA B27 (12.2%).

**Conclusion:**

This study demonstrated that male/female ratio and mean age at onset were comparable to the results of previous studies in Iran. Significant associations between HLAB5 and clinical manifestations underline the pivotal role of genetic factors in BD.

## INTRODUCTION

Behçet’s Disease (BD), also known as “silk road disease,” is a systematic disease with various clinical manifestations classified as a systemic variable vessel vasculitis. Clinically, BD presents with oral and genital aphthous ulcers, skin lesions, ocular, articular, neural, and gastrointestinal manifestations.^1^ Regarding geographical distribution, BD has a higher prevalence in Mediterranean countries, Far East, and the Middle East, while being less prevalent in America, Oceania, and sub-Saharan Africa.^2^ Current evidence shows that the highest prevalence of BD belongs to Turkey (20 to 420 patients per 100,000 population) and Iran (80 to 100 patients per 100,000 population).^3^ Limited data is available regarding the incidence of the disease, though the incidence of 0.57 new cases per 100,000 population has been reported.^4^ BD is associated with intermittent episodes of relapse and recovery; each episode may involve one or several organs and last for totally variable periods.^5^ Disease activity is higher in the first years of disease diagnosis, which declines gradually to complete recovery in 60% of patients on average 20 years after disease diagnosis.^6^

Previous studies suggest that the mean age of disease onset is 26.2 years in Iran. The male/female ratio of disease prevalence was reported to be 1.19 in Iran.^7^ It is worth mentioning that, contradictory results have been reported in case-series studies in terms of age and sex distribution. For instance, male/female ratios of 3.67 and 4.9 have been reported for prevalence in Russia and Kuwait respectively, while this ratio is <1 in some countries such as Spain and Sweden. Similarly, a wide range of results have been reported regarding the age of disease onset; for instance, it has been shown to be 40 years in Brazil and 20.8 years in Ireland.^6,7^

Mucocutaneous lesions and ocular involvement are among the common manifestations of BD. Oral aphthous ulcer is the most common manifestation among patients,^6^ observed in 97% of Iranian patients. Moreover, genital aphthous ulcer has been reported in 65% of cases.^7^

Pseudo-folliculitis and erythema nodosum are the two common skin manifestations of BD. Skin manifestations have been reported in 66% of patients with BD in Iran.^7^

Ocular involvement that is seen in 40% of BD patients in Iran is another common and important manifestation of the disease with anterior uveitis being the most common type of which. The ocular lesions are among the most important morbidities of BD patients that even can lead to blindness.^7^

Considering what is stated above, BD is associated with considerable heterogeneity among the different populations in terms of clinical manifestations, demographic factors, severity, and frequency of disease recurrence, treatment response, and disease outcomes.^8^ Thus, studying the clinical manifestations and characteristics of the disease, which may be unique to the geographical area, is very helpful in understanding the aetiology of the disease and its management. On the other hand, since Iran is among the countries with the highest prevalence of BD, the present study aims to investigate the clinical manifestations of BD.

## MATERIALS AND METHODS

### Study design and participants

In a retrospective, cross-sectional study the medical records of BD patients in the Rheumatology clinics of Vali-E-Asr Hospital, Zanjan, and Shariati Hospital, Tehran were investigated, and their information was recorded. Rheumatology Research Centre (RRC) in Shariati Hospital, an educational hospital affiliated with Tehran University of Medical Sciences, is Iran’s first and most important Rheumatology research centre. Rheumatology clinic of Vali-E-Asr Hospital is the only referral clinic of rheumatology in Zanjan Province, affiliated with Zanjan University of Medical Sciences. The study protocol was approved by research ethics committee of Zanjan University of Medical Sciences (IR.ZUMS.REC.1398.186). All patient information was kept confidential.

### Inclusion and exclusion criteria

Inclusion criteria was considered the definite diagnosis of BD by a rheumatologist according to International Criteria for Behçet’s Disease 2014 (ICBD).^9^ The criteria include six items of vascular manifestations, oral aphthous, genital aphthous, skin manifestations (erythema nodosum, pseudo-folliculitis), ocular manifestations (anterior uveitis, posterior uveitis, and retinal vasculitis), CNS involvement, and positive pathergy test. In these criteria, all items get one score except for oral aphthous, genital aphthous, and ocular involvement, getting two scores. Patients with a score of 4 or higher are diagnosed with BD. Patients with missing data in their medical records were excluded from the study. It is worth mentioning that ocular, neural, and cutaneous involvements were independently appraised and diagnosed by an ophthalmologist, neurologist, and dermatologist, respectively.

### Study variables

Age, sex, the delay between the onset of symptoms and diagnosis, clinical manifestations (mucosal involvement, skin involvement, ocular involvement, articular involvement, gastrointestinal involvement, vascular involvement, epididymitis, neurologic symptoms, lung involvement), HLA B27, HLA B51, HLA B5, haematuria, proteinuria, leukocyturia, Erythrocyte Sedimentation Rate (ESR), and pathergy phenomenon were extracted from patients’ medical records.

### Statistical analysis

Numerical data were reported as mean ± standard deviation (SD), and categorical data were reported as frequency (percentage). Chi-square and Fisher’s exact tests were used to compare categorical data between two groups, and independent samples t-test was used to compare means of numerical data between two groups. The significance level in all analyses was considered <0.05. SPSS software v.23 was used for statistical analysis.

## RESULTS

A total of 188 medical records were included in the study consisting of 112 (59.6%) men and 76 (40.4%) women (Male/female ratio = 1.47).

**Figure 1.**
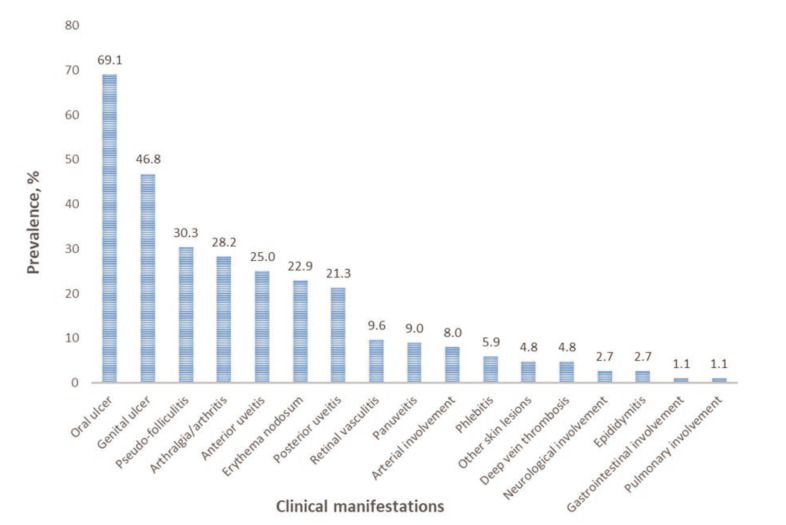
Prevalence of clinical manifestations among patients.

The age at onset was 27.98 ± 10.47 years, and the mean delay between the onset of symptoms and diagnosis was 5.70 ± 7.16 years. The most common clinical manifestation was found to be mucosal involvement (85.1%), followed by the ocular lesions (55.3%) and skin manifestations (44.7%). Additionally, lung and gastrointestinal involvements showed the least prevalence (1.1%, two patients) (**Table 1**, **Figure 1**).

**Table 1.** The clinical manifestations and basic characteristics of the patients.

**Variable**	**Mean ± SD / N (%)**
**Age, years**	27.98 ± 10.47
**Gender, male**	112 (59.6)
**Oral ulcer**	130 (69.1)
**Genital ulcer**	88 (46.8)
**Ocular involvement**	104 (55.3)
**Anterior uveitis**	47 (25.0)
**Posterior uveitis**	40 (21.3)
**Panuveitis**	17 (9.0)
**Retinal vasculitis**	18 (9.6)
**Skin lesions**	84 (44.7)
**Erythema nodosum**	43 (22.9)
**Pseudo-folliculitis**	57 (30.3)
**Other skin lesions**	9 (4.8)
**Arthralgia/arthritis**	53 (28.2)
**Gastrointestinal involvement**	2 (1.1)
**Neurological involvement**	5 (2.7)
**Vascular involvement**	28 (14.9)
**Deep vein thrombosis**	9 (4.8)
**Phlebitis**	11 (5.9)
**Arterial involvement**	15 (8.0)
**Epididymitis**	5 (2.7)
**Pulmonary involvement**	2 (1.1)

**Figure 2.**
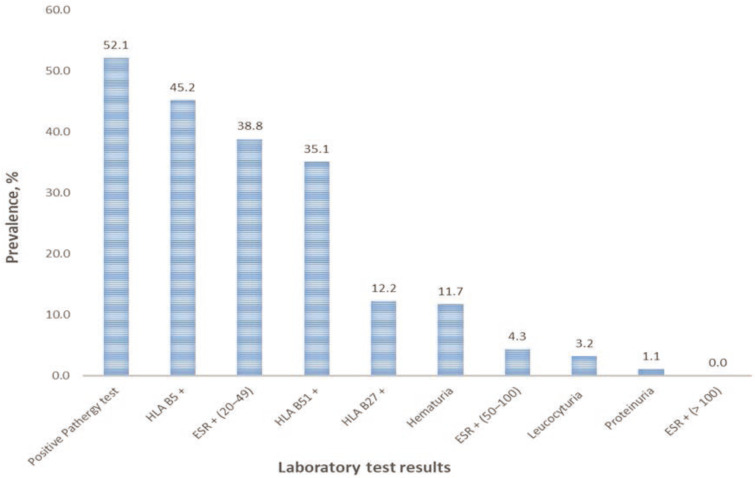
Prevalence of laboratory test results among patients.

The Pathergy phenomenon was observed in 98 patients (52.1%). Moreover, 45.2% had positive HLA B5, followed by HLA B51 (35.1%) and HLA B27 (12.2%) (**Table 2**, **Figure 2**).

**Table 2.** Laboratory test results.

**Variable**	**N (%)**
**Positive Pathergy test**	98 (52.1)
**HLA B51 +**	66 (35.1)
**HLA B5 +**	85 (45.2)
**HLA B27 +**	23 (12.2)
**ESR +**	81 (43.1)
**20–49**	73 (38.8)
**50–100**	8 (4.3)
**> 100**	0 (0)
**UA**	
**Haematuria**	22 (11.7)
**Leukocyturia**	6 (3.2)
**Proteinuria**	2 (1.1)

VDRL: Venereal disease research laboratory test, UA: urinalysis, ESR: Erythrocyte Sedimentation Rate, HLA: Human Leukocyte Antigen

A Chi-Square test was performed to determine the relationship between clinical manifestations and gender. There was a significant relationship between skin lesions and gender, X^2^ (1, N=188) = 8.86, p = 0.003. The results suggest that there was 2.5 times greater likelihood of representing skin lesions if the patients were male vs. female (OR: 2.500, 95% CI: 1.358 to 4.608).

No significant relationship was found between other variables and gender (All, P≥0.05) (**Table 3**).

**Table 3.** Gender of patients with Behçet’s disease according to the clinical manifestations and test results.

	**Male (n = 112)**	**Female (n = 76)**					
**Variables**	**n**	**%**	**n**	**%**	**OR**	**95% CI**	**P-value**
**Oral ulcer**							
**Yes**	79	70.5	51	67.1	0.852	0.455, 1.596	0.617
**No**	33	29.5	25	32.9			
**Genital ulcer**							
**Yes**	58	51.8	30	39.5	0.607	0.336, 1.096	0.097
**No**	54	48.2	46	60.5			
**Ocular involvement**							
**Yes**	58	51.8	46	60.5	1.428	0.791, 2.577	0.237
**No**	54	48.2	30	39.5			
**Skin lesions**							
**Yes**	60	53.6	24	31.6	2.500	1.358, 4.608	0.003^*^
**No**	52	46.4	52	68.4			
**Arthralgia/arthritis**							
**Yes**	34	30.4	19	25.0	0.765	0.396, 1.475	0.423
**No**	78	69.6	57	75.0			
**Deep vein thrombosis**							
**Yes**	4	3.6	5	6.6	1.901	0.494, 7.323	0.489
**No**	108	96.4	71	93.4			
**Phlebitis**							
**Yes**	6	5.4	5	6.6	-	-	0.759
**No**	106	94.6	71	93.4			
**Arterial involvement**							
**Yes**	11	9.8	4	5.3	0.510	0.156, 1.666	0.258
**No**	101	90.2	72	94.7			
**Neurological involvement**							
**Yes**	4	3.6	1	1.3	0.360	0.039, 3.285	0.650
**No**	108	96.4	75	98.7			
**Gastrointestinal involvement**							
**Yes**	2	1.8	0	0	-	-	0.516
**No**	110	98.2	76	100			
**Pulmonary involvement**							
**Yes**	2	1.8	0	0	-	-	0.516
**No**	110	98.2	76	100			
**Positive Pathergy test**							
**Yes**	50	44.6	40	52.6	1.378	0.768, 2.472	0.282
**No**	62	55.4	36	47.4			
**HLA B51 +**							
**Yes**	43	38.4	23	30.3	1.436	0.772, 2.670	0.252
**No**	69	61.6	53	69.7			
**HLA B5 +**							
**Yes**	53	47.3	32	42.1	1.235	0.687, 2.222	0.481
**No**	59	52.7	44	57.9			
**HLA B27 +**							
**Yes**	10	8.9	13	17.1	0.475	0.197, 1.148	0.093
**No**	102	91.1	63	82.9			
**ESR +**							
**Yes**	47	42.0	34	44.7	1.120	0.622, 2.015	0.706
**No**	65	58.0	42	55.3			
**UA +**							
**Yes**	17	15.2	13	17.1	1.153	0.524, 2.539	0.723
**No**	95	84.8	63	82.9			

VDRL: Venereal disease research laboratory test, UA: urinalysis, ESR: Erythrocyte Sedimentation Rate, HLA: Human Leukocyte Antigen

* P < 0.05

**Figure 3.**
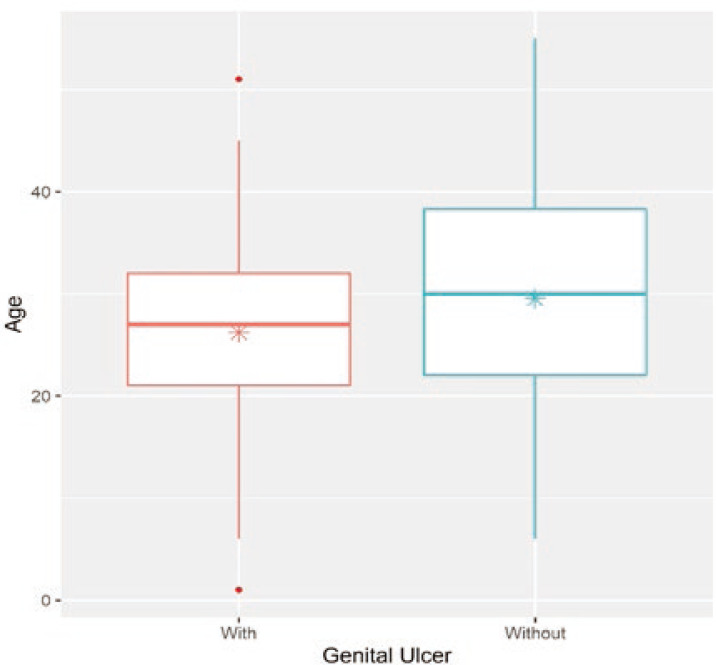
Boxplot of age by genital ulcer.

Patients with genital ulcer (mean ± SD, 26.20 ± 10.13) were found to be significantly younger than those without genital ulcer (mean ± SD, 29.54 ± 10.90), t(186) = −2.162, P = 0.032 (**Figure 3**).

No significant difference was discovered between other variables in view of the patients’ age (All, P≥0.05).

The proportion of subjects who represented clinical manifestations did not differ by the delay between the onset of symptom and diagnosis (<10 or >10 years) (All, P≥0.05).

Also, a chi-square test was performed to examine the relationship between the results of laboratory tests and clinical manifestations.

**Figure 4.**
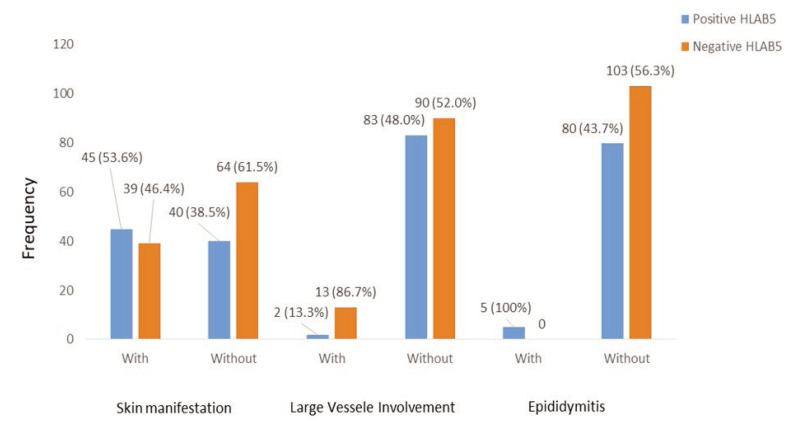
Bar chart of HLAB+ prevalence according to skin manifestations, arterial involvement, and epididymitis.

There was a significant association between HLA-B5+ and skin manifestations, X^2^(1, N = 188) = 4.283, P = 0.038). The odds were nearly 1.5 times greater to represent skin manifestations if patients had HLA-B5+ (OR: 1.607, 95%CI: 1.102 to 3.311) (**Figure 4**).

The relationship between large vessel involvement and HLA-B5+ was significant, X^2^ (1, N = 188) = 6.688, P = 0.01. There was approximately 6 times more likelihood to get diagnosed with arterial involvement if the patients tested negative for HLA-B5. (OR: 5.994, 95%CI: 1.313 to 27.361) (**Figure 3**).

A Fisher’s Exact Test revealed that the relationship between epididymitis and HLA-B5+ was also significant (P = 0.018) (**Figure 3**).

Further analysis showed that there were no significant associations between other laboratory tests (HLA-B27, HLA-B51 ESR, urinalysis, Pathergy) and clinical manifestations (All, P≥ 0.05).

## DISCUSSION

BD, a rare disease with a worldwide incidence, is specifically more prevalent in South-East Asia (ie, China, Japan, and Korea), Middle-East (Iran) and some Mediterranean countries, including, Turkey, and Greece.^10^

However, BD is known to be of unknown origin, various associations with genetic factors have been found so that, it would be considered a genetically complex disease.^11,12^ That BD is more prevalent particularly in some specific geographic regions and ethnic groups, implies the strong role of genes. It has been confirmed that there are established associations between BD and HLA-B51, the interleukin-10 (IL10) variant and the one located between the interleukin-23 receptor (IL23R) and interleukin 12 receptor β2 (IL12RB2) genes.^12^

Evidence suggests a strong association between HLA-B5 and HLA-B51 with BD particularly among specific ethnicities, for example, when it comes to some Middle Eastern and Asian countries, HLA-B51 has been reported to be present in 40–70% of which, however, it exists in only 13% of patients in Europe and North America.^11,13^

Davatchi et al. investigated 7187 patients with BD in their study, in which the highest frequency was reported to be for HLA-B5+ (54%), followed by HLA-B51+ and HLA− B27+.^3^

The relationship between HLA-typing and the clinical manifestations of BD was investigated in this study. The present study suggested a significant relationship between HLA-B5+ with skin manifestations, epididymitis and arterial involvement.

A meta-analysis has shown a strong positive correlation between positive HLA-B5/51 and genital ulcers, ocular involvement, and skin manifestations.^14^ As evidenced by Shenavandeh et al. there is a significant relationship between positive HLA-B51 and ocular involvement in patients with BD.^15^

A literature review has demonstrated that BD is more common among men than women so that, the male-to-female ratio was reported > 1 in a majority of studies (1.47 in the present study), although it has been remarkably high in some countries like Egypt (5.37), Algeria (4.27), Russia (3.67), Saudi Arabia (3.40), and Iraq (3.10).^16^

There has been discrepancies between studies in terms of gender-related differences of BD. To be specific, our findings revealed a higher frequency of skin manifestations among male than female patients. Even so, in a large cohort of Chinese patients there was not any significant association between gender and skin manifestations. Another example shows the conflict with regard to neurological manifestations, indicating a male predominance in Turkey versus a female predominance in Iran. Disparities in ethnic groups, geographic regions, study designs, or diagnostic criteria may be a reason for this.^17^

Chen et al. stated that however, the odds of being diagnosed with vascular BD is greater among male patients, there has been a similar incidence rate between males and females in the postmenopausal age. Thus, gender or hormones might protect female BD patients from vascular involvement.^18^

A significant relationship has been reported between genital aphthous, skin manifestations (pseudo-folliculitis and erythema nodosum), as well as ocular involvement with gender so that, genital aphthous ulcer has been shown to be more prevalent among females, while, ocular involvement and skin manifestations has been more common among males.^16^ Another study has reported that vascular involvement, ocular involvement, cardiac involvement, neurologic involvement, and pathergy phenomenon are more common among males than females.^19^

A study on 20 patients with BD in Pakistan showed no significant difference between men and women in terms of clinical manifestations,^1^ which may be due to the limited sample size. However, significant associations have been reported between gender and clinical manifestations in studies with high sample size.^3,16,19^

The most common clinical manifestations were mucosal lesions (oral and genital aphthous), skin lesions, ocular involvement, and articular involvement, respectively. Yet, ocular involvement was more common than skin manifestation in the present study. Additionally, their study showed, Pathergy phenomenon were observed in 52.3% of the patients, which is consistent with the findings of our study.^3^ Abnormal ESR was less common in our study than in the Davatchi et al. study (431% compared with 52.8%). Recently, ESR and CRP were mostly used to assess the clinical activity of Behcet’s disease, though the relationship between ESR and the clinical activity of the disease has been controversial. Studies conducted in Iran show that elevated levels of ESR are mostly seen in patients with genital lesions. Other studies have suggested that elevated levels of ESR and CRP are associated with newly-formed erythema nodosum, superficial thrombophlebitis, and articular involvement.^6,20^

A study on patients with BD in the United States indicated that the most common clinical manifestations were mucosal lesions (100%), ocular involvement (62%), skin manifestations (85%), and articular involvement (46%).^21^

Moreover, in a study on 1996 patients with BD in China, the most common clinical manifestations were oral aphthous ulcer (98.4%), genital aphthous ulcer (76.3%), erythema nodosum and pseudo-folliculitis (69%), ocular involvement (34.8%), and articular involvement (30%).^19^

We demonstrated that patients with genital ulcer were significantly younger than those without genital ulcer. Li et al. inferred that pseudo-folliculitis and genital ulcer were significantly more frequent in younger BD patients.^17^

Melikoğlu et al. showed that oral ulcers and arthralgia were more common in patients below 30 years of age, while no significant difference was observed between age groups regarding disease activity.^22^ In the present study, the mean age of patients with BD at onset was 27.98 ± 10.47 years. Studies in different countries have reported ages ranging between 26 and 35.7 years.^7^ Davachi et al. reported the age of 28.3 years, which is in line with the findings of our study.^3^ A main reason for such age range might be due to the disease’s auto-inflammatory nature that usually occurs more commonly at young ages.^7^

## LIMITATIONS

The present study has some limitations. First, we gathered data from patients’ medical records which may not be adequately accurate since they were mostly recorded for non-research purposes. However, we only included patients with complete medical records (clinical manifestations and laboratory tests). It is recommended to conduct prospective and case-control studies to be able to evaluate and compare the clinical manifestations of the disease accurately. The low sample size is another important limitation of the current study, making the analysis relatively underpowered to detect significant effects of the variables.

## CONCLUSION

In summary, this study found that male/female ratio and the mean age of patients at onset was very close to the results of previous studies in Iran. We failed to show significant associations between gender and clinical manifestations except for skin involvement. In agreement with previous data this study also shed light on significant links between HLAB+ and some of the clinical manifestations of BD. These findings emphasize the role of genes and the fact that they might be useful for clinicians to predict the probability of possible approaching symptoms in patients. The authors of the present study recommend conducting longitudinal studies to investigate more predisposing genetic factors of BD in terms of clinical manifestations of the disease. Additionally, there is a need to evaluate some certain aspects of the burden of the disease, eg, the use of immunosuppressive agents and irreversible organ damage which could be assessed using BD Overall Damage Index (BODI).
